# Radiotherapy for Langerhans Cell Histiocytosis of Bilateral Eyelids

**DOI:** 10.7759/cureus.474

**Published:** 2016-02-02

**Authors:** James Leveson, Jean-Marc Bourque, Jelena Lukovic, A. Rashid Dar

**Affiliations:** 1 King's College London; 2 Department of Radiation Oncology, London Regional Cancer Program

**Keywords:** ophthalmology, local, radiation therapy, lch, histiocytosis x, langerhans cell histiocytosis

## Abstract

Langerhans cell histiocytosis (LCH) is a rare disorder with numerous clinicopathological variants with differing clinical courses, treatment methods, and prognoses. We report one patient with atypical LCH of the bilateral lower eyelids and subsequent successful treatment with local radiation therapy.

## Introduction

Langerhans cell histiocytosis (LCH) is uncommon in the general population, and there is a paucity of literature on this topic. The disease can present in various forms and local treatment varies widely depending on the affected site. We present a case of atypical LCH in which numerous initial local treatments were unsuccessful. Finally, we demonstrate the effectiveness of radiotherapy as a treatment of LCH, significantly improving the patient's quality of life.

## Case presentation

Informed patient consent was obtained for this patient's treatment.

In November 2007, a 37-year-old white male presented to his family physician complaining of bilateral lower eyelid growths that had progressed from the left to the right side. He was initially diagnosed as chalazion with blepharitis and treated with local steroid injection without improvement. In July 2008, a skin biopsy specimen of the left lower lid (tarsal conjunctival area) showed high cellularity, Langerhans cells (LCs) with nuclear grooves and intranuclear inclusions, numerous eosinophils, lymphocytes, macrophages, neutrophils, and giant cells. There was no extra-orbital disease; orbital CT revealed no gross abnormalities and MRI of the head showed the craniocervical anatomy to be normal. Bone marrow aspiration revealed normal cellular trilineage hematopoietic marrow. Further investigations, including chest x-ray and routine blood work, revealed no other abnormalities. Review of the patient presentation and histology supported the diagnosis of LCH.

**Figure 1:**
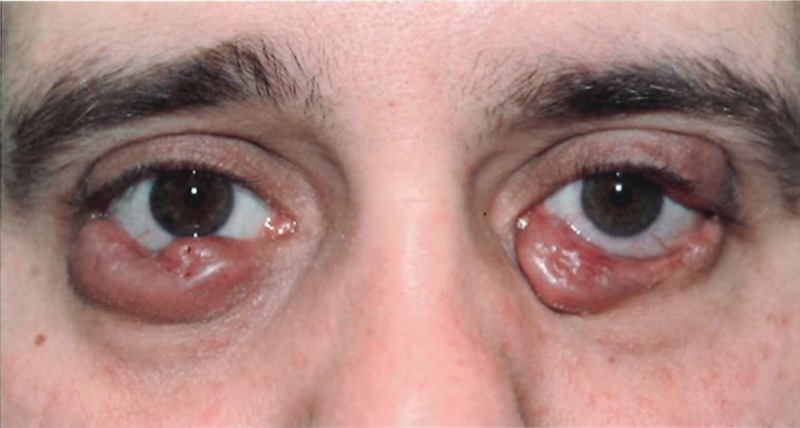
Before radiation treatment (February 2012) Prior treatments included surgical debulking and periodic steroid injections.

Once this diagnosis was confirmed, the patient underwent surgical debulking with subsequent periodic corticosteroid injections. This therapy did not achieve adequate control, and the disease subsequently progressed, with an invasion of the cilia bilaterally and the right tarsus muscle (Figure 1). No visual disturbances were found.

**Figure 2:**
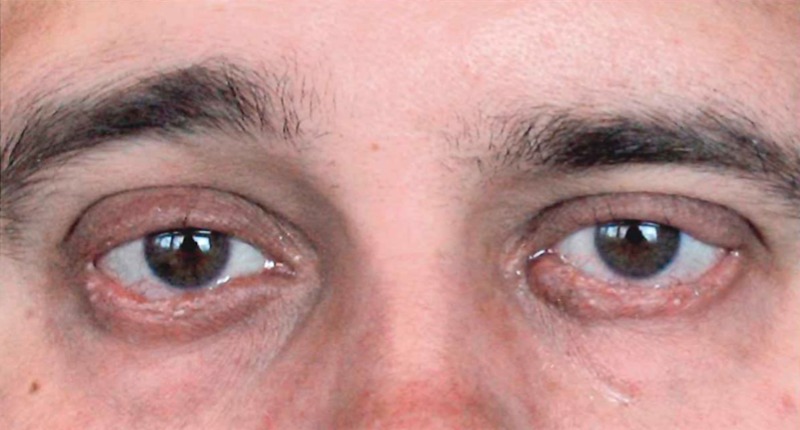
After radiation treatment (April 2012) 7 x 300 cGy exposure = 2100 cGy total exposure.

In February 2012, the patient was treated with a dose of 2,100 cGy in seven fractions utilizing 6 MeV electrons with a 0.5 cm bolus over the skin on each eyelid. The patient tolerated the therapy well with madarosis and exophthalmia being his main complications, for which he used TobraDex drops. At his three month follow-up by the radiation oncology, ophthalmology, and plastic surgery teams, the patient was noted to have a near-complete response with a remarkable cosmetic outcome. There was some residual whitish area on the left outer superior margin of the eyelid, corresponding to necrosis, but no evidence of residual disease (Figure 2). His vision was 20/25 bilaterally and the corneas were unremarkable. Unfortunately, no further follow-up information is available due to non-attendance.

## Discussion

Histiocytes are cells belonging to the reticuloendothelial system and include monocytes/macrophages, dermal/interstitial dendritic cells, and Langerhans cells (LC). They are hypothesised to arise from a common CD-34 positive progenitor cell within the bone marrow, which further differentiates along two major pathways, namely, CD-14 positive cells or CD-14 negative cells. CD-14 positive cells differentiate into macrophages and dendritic cells, and CD-14 negative cells become LCs. LCs are usually characterised by the presence of Birbeck granules, which are organelles thought to be involved in antigen internalisation [1-2].

The histiocytoses encompass a heterogeneous group of proliferative disorders distinguished by the accumulation of histiocytes and other immune effector cells within various tissues. Currently, they are classified into three classes based on the predominant cell type present [3].

LCH, a Class I histiocytosis, is a rare disease with an estimated incidence of 4.0-5.4 cases per million children per year [4]. It affects patients from the neonatal period through to adulthood, although it appears to be more common amongst children. Robust epidemiological data is difficult to attain in the literature due to the rarity of the disease; however, it has been observed that it is more common among whites. So far, there has been minimal progress in assessing the cause of LCH but some associations with thyroid disease have been demonstrated.

The pathophysiology of LCH remains elusive [5]. Research understanding of LCH has been greatly improved by analysing primary human material with advanced genomic technologies. LCH may arise from an earlier precursor cell given that cell gene expression is different from those in normal Langerhans cells (LCs), although this is not incongruous with the hypothesis that LCs are the cell of origin. Langerhans cells have been demonstrated to be clonal, and recent advances have identified activating *BRAF* mutations, which strongly suggests LCH is a neoplastic disease. However, the frequently benign course of the disease and a prominent inflammatory component suggest that LCH may not be a neoplasm. 

LCH has a wide range of clinicopathological presentations [6]. It may involve almost any organ system, but the frequency and extent of disease are often age-dependent. In neonates and children under four years, it most frequently presents as a multiorgan disease (51% to 71%) [7-11]. Most retrospective studies in adults show that adults only have involvement of a single organ system (69% to 72%) [7-15]. However, a recent large multinational retrospective study contradicts these reports, attributing the higher observed systemic disease (68.9%) to improved recognition and diagnosis [15]. When a single organ system is involved, LCH is most often characterised by bone lesions (52%), lung lesions (40%), or skin lesions (7%), but central nervous system involvement has also been described.

Diagnosing LCH involves identification of the characteristic clinical features as well as a corroboration of the histological (uncontrolled clonal proliferation of normal human mononuclear phagocytes called Langerhans cells) and immunohistochemical results (lesional cells staining positive for S-100 and CD1a) [16-17]. Other pathognomonic markers are being sought. Investigations often include full skeletal radiographic survey and chest radiography but more specific investigations, such as bone marrow evaluation, may be required. In our patient, the similarity of the presentation to more common ophthalmological disease initially led to misdiagnosis and a delay in treatment.

Treatment protocol for LCH depends on a number of variables [3, 18-19]. Treatment of multisystem disease is based on the LCH-III protocol [20]. However, this discussion will explore treatment options for the single system (cutaneous) disease. Whilst patients with the limited cutaneous disease usually require no therapy, steroids are often utilised. Topical nitrogen mustard or psoralen combined with ultraviolet A (PUVA) treatment are viable second-line options [21]. 

Radiation therapy (RT) is considered an appropriate option for LCH, particularly when local control is challenging in recurrent and progressive lesions [22]. In a recent retrospective study with 80 patients, RT was shown to be an extremely well-tolerated option, with even low RT doses demonstrating sufficient control. In this study, RT was carried out with a median cumulative dose of 15 Gy (range: 3 - 50.4 Gy) and a median 2 Gy dose per fraction (range: 1.8 - 3 Gy). Additionally, radiogenic side and late effects ≥ EORTC/RTOG Grade 2 were not observed, and there were no treatment-related deaths or secondary malignancies reported after a median follow-up of  54 months (range: 9 – 134 months) [23].

Other studies also support the efficacy of RT in the treatment of LCH [24-28]. One study with twenty-two LCH patients, where 56 sites of LCH were irradiated (40 bone, 16 soft tissue), achieved local control in 82% of these sites utilising a median dose of 900 cGy for bone (range: 600 – 1,500) and 1,500 cGy for soft tissue sites (range: 600 – 2,600). Another particular case report describing a paediatric orbital manifestation of LCH also achieved remission following external radiotherapy (1,500 cGy total dose in three fractions) [29].

## Conclusions

LCH is a rare disease with multiple clinicopathological variants. Treatment regime varies depending on multiple factors. We have described a case where the use of localised RT successfully achieved a complete response with a remarkable cosmetic outcome. This adds to the evidence supporting RT as an effective, safe, and simple treatment option in recurrent and progressive lesions.
